# Crystal structure and Hirshfeld surface analysis of 3-(bromo­meth­yl)-2-[1,2-di­bromo-2-(6-nitro­benzo[*d*][1,3]dioxol-5-yl)eth­yl]-1-(phenyl­sulfon­yl)-1*H*-indole chloro­form 0.585-solvate

**DOI:** 10.1107/S2056989023007120

**Published:** 2023-08-17

**Authors:** Nagaraj Achyuta, Somarathinam Kanagasabai, Pavunkumar Vinayagam, Arasambattu K. Mohanakrishnan, Namasivayam Gautham, Krishnasamy Gunasekaran

**Affiliations:** a CAS in Crystallography and Biophysics, University of Madras, Chennai, India; b Department of Organic Chemistry, University of Madras, Chennai, India; University of Aberdeen, United Kingdom

**Keywords:** crystal structure, synthesis, 1-(phenyl­sulfon­yl)-1*H*-indole, Y—*X*⋯π inter­actions, hydrogen bonding, Hirshfeld surface analysis

## Abstract

The title indole derivative, C_24_H_17_Br_3_N_2_O_6_S, crystallizes with a partial occupancy [0.585 (4)] CHCl_3_ solvent mol­ecule. The dihedral angles between the indole ring system and pendant nitro­benzodioxolane rings system and phenyl­sulfonyl ring are 4.81 (14) and 72.24 (19)°, respectively. In the crystal, the indole mol­ecules are linked to each other and to the chloro­form mol­ecule by weak C—H⋯O, C—H⋯Cl, C—H⋯π, C—Br⋯π and C—Cl⋯π and aromatic π–π stacking inter­actions. A Hirshfeld surface analysis was carried out and the inter­molecular contacts with the most significant contributions are H⋯O/O⋯H (24.3%), H⋯H (18.4%), Br⋯H/H⋯Br (16.8%) and C⋯H/H⋯C (8.4%).

## Abstract 2

The title indole derivative crystallizes with a partial occupancy [0.585 (4)] CHCl_3_ solvent mol­ecule. The dihedral angles between the indole ring system and pendant nitro­benzodioxolane rings system and phenyl­sulfonyl ring are 4.81 (14) and 72.24 (19)°, respectively. In the crystal, the indole mol­ecules are linked to each other and to the chloro­form mol­ecule by weak C—H⋯O, C—H⋯Cl, C—H⋯π, C—Br⋯π and C—Cl⋯π and aromatic π–π stacking inter­actions.

## 1. Chemical context

Derivatives of indole have been reported to exhibit anti­bacterial (Okabe & Adachi, 1998 ▸) and anti­tumour (Schollmeyer *et al.*, 1995 ▸) activities. *N*-Substituted indole derivatives have been found to exhibit anti­oxidant properties (Ölgen & Çoban, 2003 ▸, 2002 ▸) and halogenated indole derivatives have demonstrated anti­bacterial and anti­fungal activity (Piscopo *et al.*, 1990 ▸). Derivatives of 1-(phenyl­sulfon­yl)indole have proven their usefulness in the synthesis of biologically active alkaloids and their related analogues, including pyridocarb­azoles, such as the anti­cancer alkaloid ellipticine, carbazoles, furo­indoles, pyrrolo­indoles, indolocarbazoles and other substituted indoles. The indole phenyl­sulfonyl moiety acts as both a protecting and activating group (Jasinski *et al.*, 2009 ▸). The phenyl­sulfonyl indole compounds have been shown to inhibit the HIV-1 RT enzyme *in vitro* and HTLVIIIb viral spread in MT-4 human T-lymphoid cells (Williams *et al.*, 1993 ▸). As part of our studies in this area, we now describe the synthesis and structure of the title mol­ecule C_24_H_17_Br_3_N_2_O_6_S·0.585CHCl_3_, which crystallized as a chloro­form solvate. 
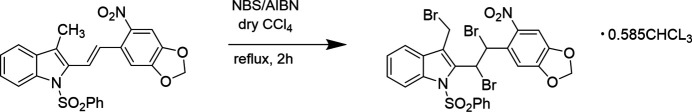


## 2. Structural commentary

**Figure 1:**
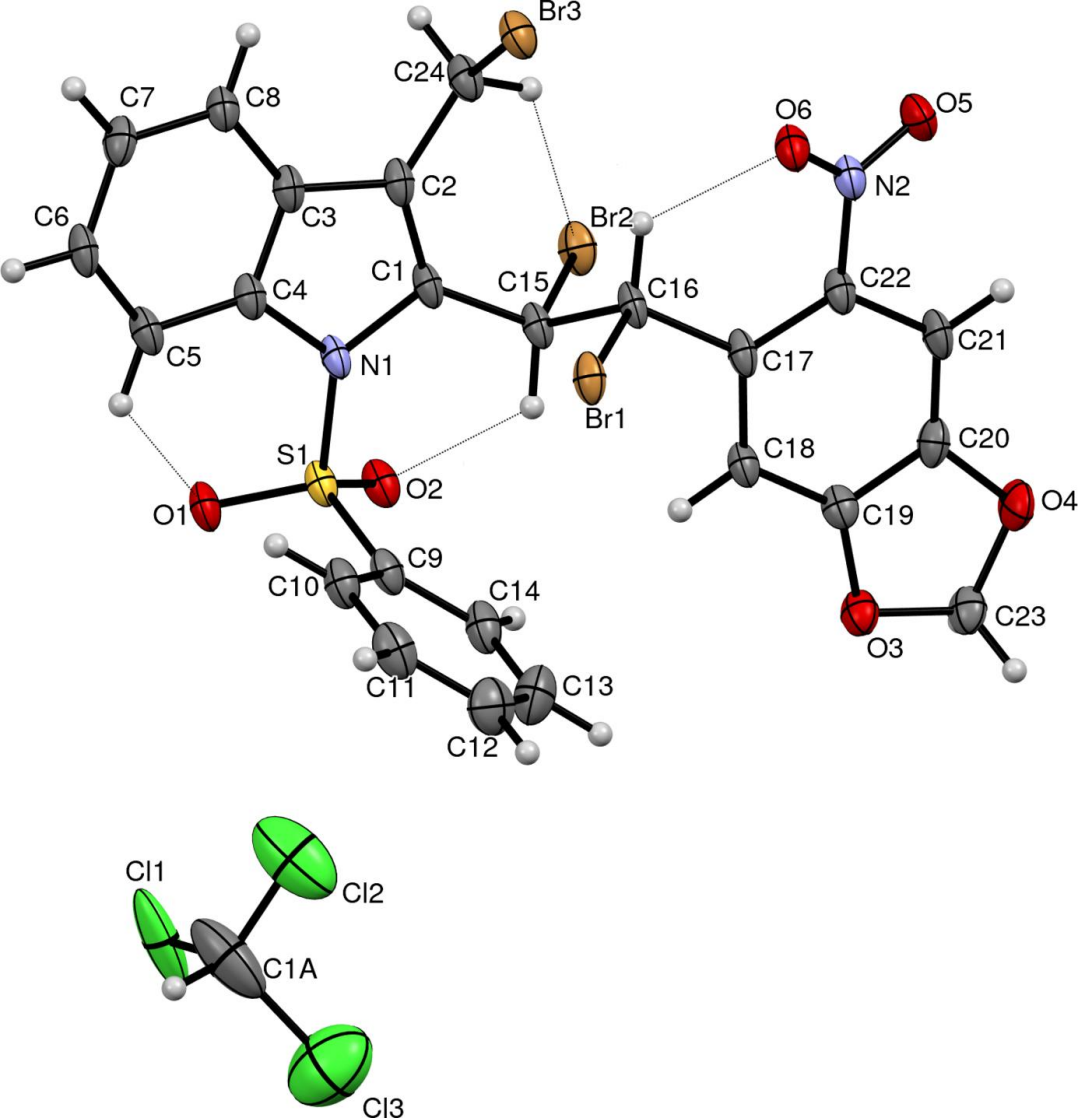
The mol­ecular structure of the title compound, with displacement ellipsoids drawn at the 50% probability level with intra­molecular hydrogen bonds shown in light blue.

The C9–C14 phenyl ring makes a dihedral angle of 72.24 (19)° with the C1–C8/N1 indole ring system (Fig. 1 ▸). The C1—C15—C16—C17 torsion angle is 175.8 (3)°. The five-member dioxolane ring (C19/C20/O4/C23/O3) adopts an envelope conformation (C23 is displaced from the plane) with pseudo rotation parameters *P* = 59.3 (17)° and τ = 10.9 (3)°, which are confirmed by the Cremer–Pople puckering parameters *Q* = 0.097 (5) Å and φ = 329 (3)°. The C1—N1 and C4—N1 bond lengths are 1.423 (5) and 1.427 (5) Å, respectively, while in the case of N atoms in planar configurations, the reported mean value is 1.355 (14) Å (Allen *et al.*, 1987 ▸). This difference is due to the electron-withdrawing nature of the phenyl­sulfonyl group attached to N1 and has been reported earlier (Palani *et al.*, 2006 ▸). During the synthesis of the title compound, bromination of the methyl group and the double bond between C15 and C16 of the 6-nitro­benzo[*d*][1,3]dioxol-5-yl)vinyl moiety occurs due to an addition reaction with Br_2_. The Br1—C16—C15—Br2 grouping is in a *trans* configuration and the torsion angle has a value of 178.14 (17)°. Intra­molecular C5—H5⋯O1, C15—H15⋯O2, C16—H16⋯O6 and C24—H24*B*⋯Br2 inter­actions (Fig. 1 ▸, Table 1 ▸) are observed.

## 3. Supra­molecular features

**Figure 2:**
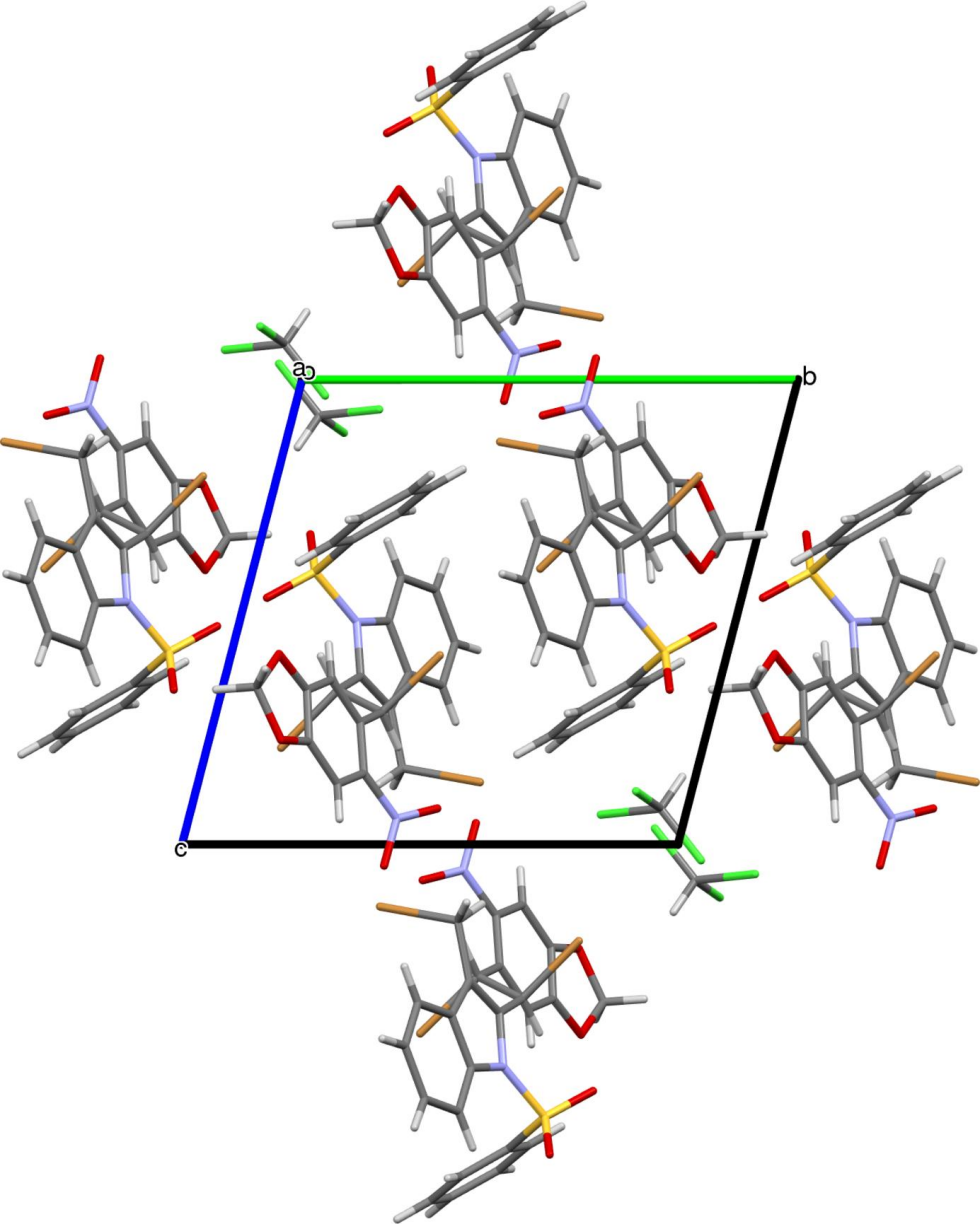
The crystal packing of the title compound viewed along the *a-*axis direction.

The extended structure exhibits weak inter­molecular hydrogen bonds including indole-to-indole C—H⋯O, indole-to-chloro­form C—H⋯Cl and chloro­form-to-indole C—H⋯O contacts (Fig. 2 ▸, Table 1 ▸). The crystal also features C—H⋯π, C—Br⋯π and C—Cl⋯π inter­actions. The C23—H23⋯*Cg*2^i^ [symmetry code: (i) *x* – 1, *y*, *z*) inter­action where *Cg*2 is the centroid of the ring C1–C4/N1 has an H⋯*Cg*2 separation of 2.94 Å with the C**—**H⋯*Cg* angle being 117°. In the case of the C—Br⋯π inter­action, the C16—Br1⋯ *Cg*4 (*Cg*4 is the centroid of the C9–C14 ring) inter­action has a Br⋯*Cg*4 distance of 3.691 (2) Å with the C**—**Br⋯*Cg* angle being 111.46 (11) °. The C1—Cl2⋯ *Cg*4 inter­action to the other face of the C9–C14 ring has a Cl⋯*Cg* distance of 3.236 (4) Å with the C**—**Cl⋯*Cg* angle being 167 (5)°.

## 4. Database survey

A search of the Cambridge Structural Database (CSD, Version 5.43, update of November 2022; Groom *et al.*, 2016 ▸) for the 3-methyl-1-(phenyl­sulfon­yl)-1*H*-indole skeleton gave four hits. The structures of 2-azido­methyl-3-methyl-1-phenyl­sulfonyl-1*H*-indole (CSD refcode AYOSIR; Karthikeyan *et al.*, 2011 ▸), 2-chloro­methyl-3-methyl-1-phenyl­sulfonyl-1*H*-indole (FUGRUV; Saravanan *et al.*, 2009 ▸) and (*E*)-2-(4-meth­oxy­styr­yl)-3-methyl-1-phenyl­sulfonyl-1*H*-indole (NUP­TUP; Umadevi *et al.*, 2015 ▸) have additional groups attached to the 3-methyl-1-phenyl­sulfonyl-1*H*-indole core. Conversely, 2-(2-{1,4-dimethyl-2-[3-methyl-1-(phenyl­sulfon­yl)-1*H*-indol-2-yl]cyclo­hex-3-en-1-yl}vin­yl)-3-methyl-1-(phenyl­sulfon­yl)-1*H*-indole tetra­hydrate (FOLGOE; Dethe *et al.*, 2014 ▸) consists of two 3-methyl-1-(phenyl­sulfon­yl)-1*H*-indole groups. The search fragment 3-bromo­methyl-1-phenyl­sulfonyl-1*H*-indole yielded one hit, 3-bromo­methyl-1-phenyl­sulfonyl-1*H*-indole-2-carbo­nitrile (TECGEO; Palani *et al.*, 2006 ▸).

## 5. Hirshfeld surface analysis

**Figure 3:**
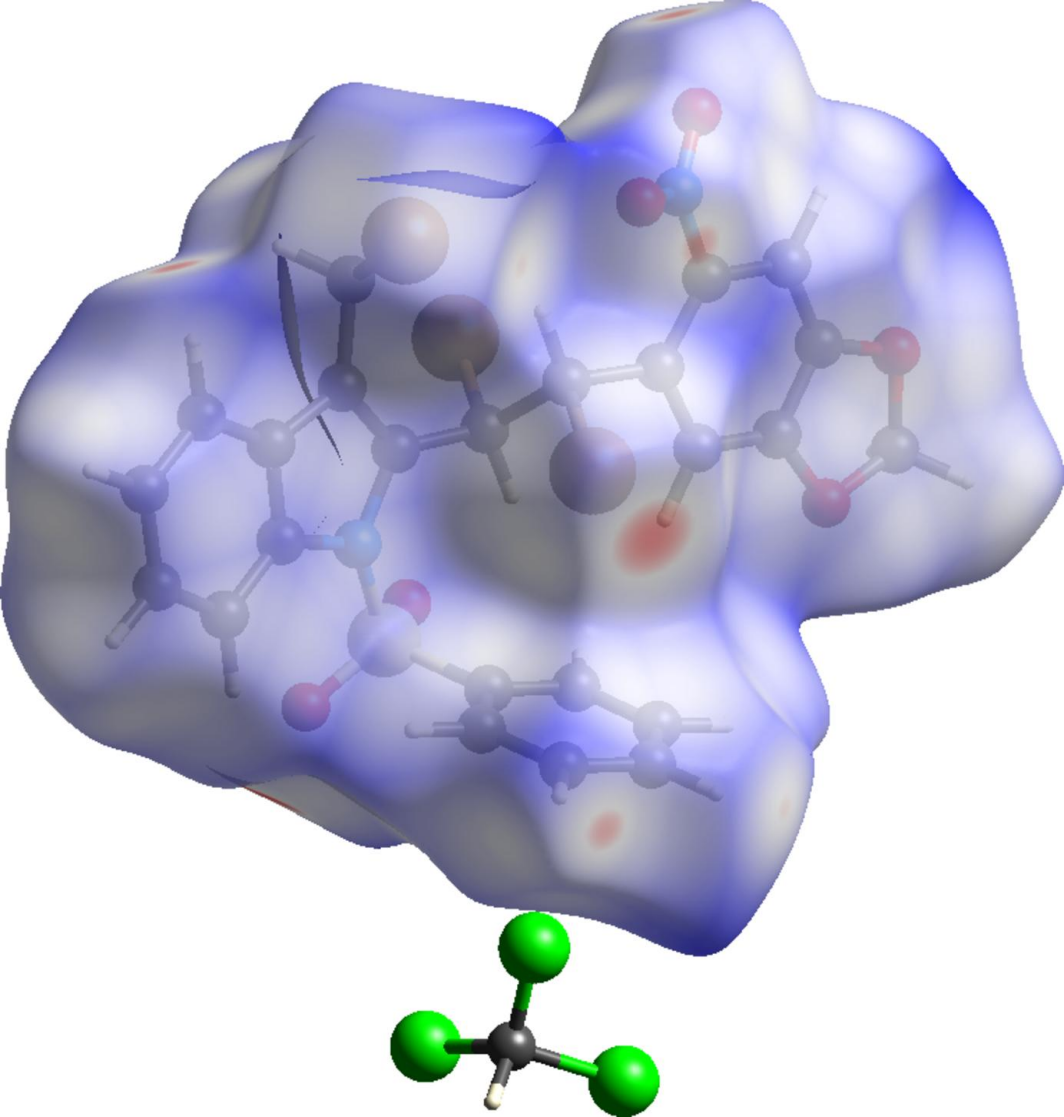
The Hirshfeld surface of the title compound mapped over *d*_norm_.

**Figure 4:**
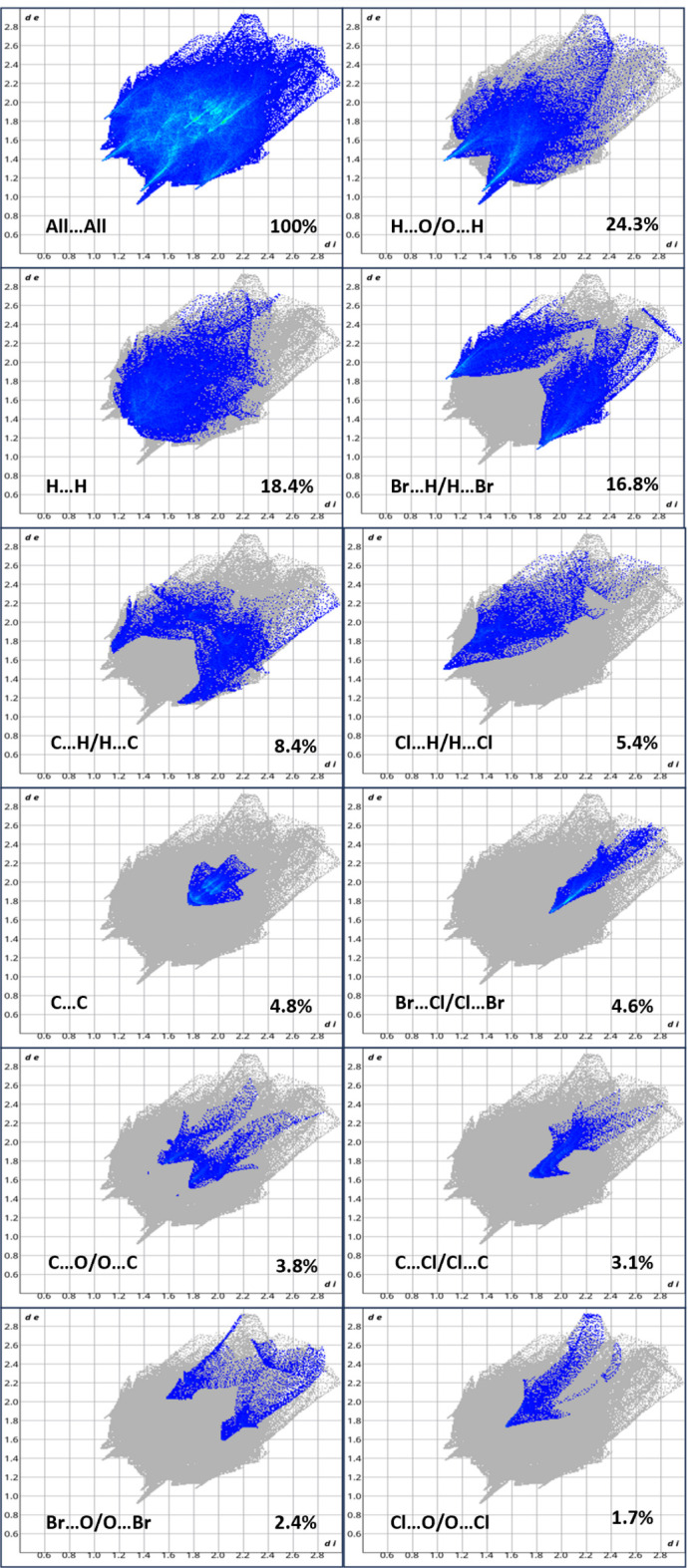
The fingerprint plots of the title compound delineated into the various labelled contacts.

The Hirshfeld surface analysis and the associated two-dimensional fingerprint plots were determined using the *Crystal Explorer 21* software (Spackman *et al.*, 2021 ▸). Fig. 3 ▸ shows the Hirshfeld surface mapped over *d*_norm_ for the title compound, where red denotes shorter contacts, blue longer contacts and the white regions indicate contacts around the van der Waals separation. The two-dimensional fingerprint plots (Parkin *et al.*, 2007 ▸) detailing the various inter­actions for the mol­ecule are shown in Fig. 4 ▸. For points on the Hirshfeld surface, *d*_i_ is the distance to the nearest atom inside and *d*_e_ is the distance to the nearest atom outside the surface. The combination of *d*_e_ and *d*_i_ in the form of a two-dimensional fingerprint plot summarizes the inter­molecular contacts in the crystal: in the title mol­ecule, the most significant inter­molecular contacts are H⋯O/O⋯H (24.3%), H⋯H (18.4%), Br⋯H/H⋯Br (16.8%) and C⋯H/H⋯C (8.4%).

## 6. Synthesis and crystallization

A solution of (*E*)-3-methyl-2-[2-(6-nitro­benzo[*d*][1,3]dioxol-5-yl)vin­yl]-1-(phenyl­sulfon­yl)-1*H*-indole (0.80 g, 1.73 mmol) and *N*-bromo­succinimide (NBS, 0.91 g, 5.19 mmol) in dry CCl_4_ (100 ml) containing a catalytic amount of azobisisobutyro­nitrile (AIBN, 50 mg) was refluxed for 2 h. The reaction mixture was cooled to room temperature. Then, the suspended succinimide was filtered off and the filtrate was concentrated *in vacuo* to obtain the crude product, which upon trituration with methanol (10 ml) gave the title compound as a bright-yellow solid. Yield: 800 mg (88%) m.p. 431–433 K. The synthesized compound was crystallized by slow evaporation using chloro­form as solvent.

## 7. Refinement

Crystal data, data collection and structure refinement details are summarized in Table 2 ▸. The hydrogen atoms were automatically added using a riding model with appropriate AFIX instructions.

## Supplementary Material

Crystal structure: contains datablock(s) I. DOI: 10.1107/S2056989023007120/hb8071sup1.cif

Structure factors: contains datablock(s) I. DOI: 10.1107/S2056989023007120/hb8071Isup3.hkl

Supporting information file. DOI: 10.1107/S2056989023007120/hb8071Isup3.cml

CCDC reference: 2279852

Additional supporting information:  crystallographic information; 3D view; checkCIF report

## Figures and Tables

**Table 1:** Hydrogen-bond geometry (Å, °)

*D*—H⋯*A*	*D*—H	H⋯*A*	*D*⋯*A*	*D*—H⋯*A*
C8—H8⋯O5^i^	0.95	2.58	3.525 (6)	170
C13—H13⋯Cl13^ii^	0.95	2.63	3.244 (8)	123
C14—H14⋯O2^iii^	0.95	2.57	3.518 (6)	172
C1*A*—H1*A*⋯O1^iv^	1.00	2.34	3.169 (15)	140
C5—H5⋯O1	0.95	2.32	2.865 (6)	116
C15—H15⋯O2	1.00	2.30	2.950 (5)	122
C16—H16⋯O6	1.00	2.16	2.832 (5)	123
C24—H24*B*⋯Br2	0.99	2.83	3.572 (4)	132

Symmetry codes: (i) 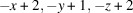 ; (ii) 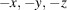 ; (iii) 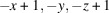 ; (iv) 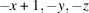 .

**Table 2:** Experimental details

Crystal data	
Chemical formula	C_24_H_17_Br_3_N_2_O_6_S·0.585CHCl_3_
*M*_r_	771.02
Crystal system, space group	Triclinic, *P* 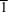
Temperature (K)	100
*a*, *b*, *c* (Å)	10.0987 (3), 12.2744 (4), 12.4842 (5)
α, β, γ (°)	99.448 (3), 110.701 (3), 100.696 (3)
*V* (Å^3^)	1377.27 (9)
*Z*	2
Radiation type	Cu *K*α
μ (mm^−1^)	8.09
Crystal size (mm)	0.09 × 0.07 × 0.04

Data collection	
Diffractometer	SuperNova, Dual, Cu at home/near, HyPix
Absorption correction	Gaussian (*CrysAlis PRO*; Rigaku OD, 2021 ▸)
*T*_min_, *T*_max_	0.679, 0.918
No. of measured, independent and observed [*I* > 2σ(*I*)] reflections	32306, 5783, 5068
*R*_int_	0.058
(sin θ/λ)_max_ (Å^−1^)	0.634

Refinement	
*R*[*F* ^2^ > 2σ(*F* ^2^)], *wR*(*F* ^2^), *S*	0.044, 0.134, 1.07
No. of reflections	5783
No. of parameters	362
No. of restraints	15
H-atom treatment	H-atom parameters constrained
Δρ_max_, Δρ_min_ (e Å^−3^)	2.45, −1.18

Computer programs: *CrysAlis PRO* (Rigaku OD, 2021 ▸), *SHELXT2018/2* (Sheldrick, 2015*a* ▸), *SHELXL2018/3* (Sheldrick, 2015*b* ▸) and *OLEX2* (Dolomanov *et al.*, 2009 ▸).

## supplementary crystallographic information

### Crystal data

**Table:**

C_24_H_17_Br_3_N_2_O_6_S·0.585CHCl_3_	*Z* = 2
*M**_r_* = 771.02	*F*(000) = 756
Triclinic, *P*1	*D*_x_ = 1.859 Mg m^−^^3^
*a* = 10.0987 (3) Å	Cu *K*α radiation, λ = 1.54184 Å
*b* = 12.2744 (4) Å	Cell parameters from 10304 reflections
*c* = 12.4842 (5) Å	θ = 3.8–77.3°
α = 99.448 (3)°	µ = 8.09 mm^−^^1^
β = 110.701 (3)°	*T* = 100 K
γ = 100.696 (3)°	Block, yellow
*V* = 1377.27 (9) Å^3^	0.09 × 0.07 × 0.04 mm

### Data collection

**Table:**

SuperNova, Dual, Cu at home/near, HyPix diffractometer	5783 independent reflections
Radiation source: micro-focus sealed X-ray tube, SuperNova (Cu) X-ray Source	5068 reflections with *I* > 2σ(*I*)
Mirror monochromator	*R*_int_ = 0.058
Detector resolution: 10.0000 pixels mm^-1^	θ_max_ = 77.8°, θ_min_ = 3.8°
ω scans	*h* = −12→12
Absorption correction: gaussian (CrysAlisPro; Rigaku OD, 2021)	*k* = −15→15
*T*_min_ = 0.679, *T*_max_ = 0.918	*l* = −15→15
32306 measured reflections	

### Refinement

**Table:**

Refinement on *F*^2^	15 restraints
Least-squares matrix: full	Hydrogen site location: inferred from neighbouring sites
*R*[*F*^2^ > 2σ(*F*^2^)] = 0.044	H-atom parameters constrained
*wR*(*F*^2^) = 0.134	*w* = 1/[σ^2^(*F*_o_^2^) + (0.0756*P*)^2^ + 3.2347*P*] where *P* = (*F*_o_^2^ + 2*F*_c_^2^)/3
*S* = 1.07	(Δ/σ)_max_ = 0.001
5783 reflections	Δρ_max_ = 2.45 e Å^−^^3^
362 parameters	Δρ_min_ = −1.18 e Å^−^^3^

### Special details

**Table:**

Geometry. All esds (except the esd in the dihedral angle between two l.s. planes) are estimated using the full covariance matrix. The cell esds are taken into account individually in the estimation of esds in distances, angles and torsion angles; correlations between esds in cell parameters are only used when they are defined by crystal symmetry. An approximate (isotropic) treatment of cell esds is used for estimating esds involving l.s. planes.

### Fractional atomic coordinates and isotropic or equivalent isotropic displacement parameters (Å^2^)

**Table:**

	*x*	*y*	*z*	*U*_iso_*/*U*_eq_	Occ. (<1)
Br1	0.58451 (5)	0.42132 (3)	0.59315 (4)	0.02570 (13)	
Br2	0.73154 (5)	0.14955 (4)	0.79206 (4)	0.02939 (13)	
Br3	1.00762 (5)	0.56830 (4)	0.86622 (4)	0.02920 (13)	
S1	0.68128 (11)	0.12626 (8)	0.41364 (8)	0.0223 (2)	
O2	0.6345 (3)	0.0430 (2)	0.4695 (3)	0.0253 (6)	
O4	0.1132 (3)	0.1454 (3)	0.7754 (3)	0.0330 (7)	
O1	0.7479 (3)	0.0973 (2)	0.3329 (3)	0.0262 (6)	
O3	0.1207 (3)	0.0964 (3)	0.5903 (3)	0.0274 (6)	
O6	0.6713 (3)	0.4960 (3)	0.9228 (3)	0.0280 (6)	
O5	0.6049 (4)	0.4185 (3)	1.0457 (3)	0.0343 (7)	
N1	0.8057 (4)	0.2367 (3)	0.5219 (3)	0.0218 (7)	
N2	0.5943 (4)	0.4196 (3)	0.9449 (3)	0.0252 (7)	
C20	0.2451 (5)	0.2022 (4)	0.7782 (4)	0.0257 (8)	
C19	0.2496 (4)	0.1717 (3)	0.6678 (4)	0.0231 (8)	
C2	0.9429 (5)	0.3497 (3)	0.7066 (4)	0.0229 (8)	
C9	0.5355 (5)	0.1832 (3)	0.3452 (4)	0.0242 (8)	
C8	1.1596 (5)	0.4418 (4)	0.6586 (4)	0.0243 (8)	
H8	1.219006	0.487579	0.736352	0.029*	
C10	0.5593 (5)	0.2662 (4)	0.2858 (4)	0.0272 (8)	
H10	0.651715	0.290181	0.281265	0.033*	
C24	1.0022 (5)	0.4044 (4)	0.8361 (4)	0.0259 (8)	
H24A	1.102550	0.395934	0.874397	0.031*	
H24B	0.939908	0.364847	0.871497	0.031*	
C16	0.6285 (4)	0.3342 (3)	0.7156 (3)	0.0215 (7)	
H16	0.708992	0.384543	0.790336	0.026*	
C15	0.6810 (5)	0.2339 (3)	0.6679 (4)	0.0221 (8)	
H15	0.598316	0.182040	0.595478	0.027*	
C21	0.3578 (5)	0.2820 (4)	0.8701 (4)	0.0275 (9)	
H21	0.354011	0.304239	0.945258	0.033*	
C7	1.2061 (5)	0.4458 (4)	0.5668 (4)	0.0277 (9)	
H7	1.298673	0.494833	0.581795	0.033*	
C4	0.9377 (4)	0.3002 (3)	0.5193 (4)	0.0227 (8)	
C3	1.0226 (4)	0.3683 (3)	0.6332 (4)	0.0228 (8)	
C17	0.4909 (5)	0.2960 (3)	0.7380 (4)	0.0224 (8)	
C22	0.4805 (5)	0.3296 (3)	0.8468 (4)	0.0232 (8)	
C18	0.3703 (5)	0.2154 (3)	0.6456 (4)	0.0232 (8)	
H18	0.372040	0.191755	0.569903	0.028*	
C23	0.0388 (5)	0.0681 (4)	0.6607 (4)	0.0278 (8)	
H23A	−0.062233	0.075806	0.623430	0.033*	
H23B	0.032817	−0.011831	0.667024	0.033*	
C6	1.1179 (5)	0.3783 (4)	0.4524 (4)	0.0272 (9)	
H6	1.151707	0.383937	0.390962	0.033*	
C1	0.8102 (5)	0.2724 (3)	0.6378 (4)	0.0228 (8)	
C5	0.9823 (5)	0.3031 (4)	0.4253 (4)	0.0261 (8)	
H5	0.923686	0.256456	0.347776	0.031*	
C11	0.4463 (6)	0.3134 (4)	0.2336 (4)	0.0345 (10)	
H11	0.460782	0.370752	0.193161	0.041*	
C14	0.4009 (5)	0.1455 (4)	0.3522 (4)	0.0296 (9)	
H14	0.386714	0.088451	0.393030	0.036*	
C12	0.3110 (6)	0.2768 (4)	0.2402 (5)	0.0390 (11)	
H12	0.233581	0.309611	0.204504	0.047*	
C13	0.2885 (6)	0.1925 (4)	0.2988 (5)	0.0397 (11)	
H13	0.195483	0.167228	0.302029	0.048*	
Cl2	0.3028 (5)	0.0547 (3)	0.0343 (3)	0.0840 (13)	0.585 (4)
Cl3	0.0526 (5)	−0.1122 (4)	−0.1145 (5)	0.1007 (15)	0.585 (4)
Cl1	0.3205 (5)	−0.1710 (3)	−0.0639 (3)	0.0828 (13)	0.585 (4)
C1A	0.247 (2)	−0.0539 (11)	−0.0772 (11)	0.079 (4)	0.585 (4)
H1A	0.257878	−0.026390	−0.145381	0.094*	0.585 (4)

### Atomic displacement parameters (Å^2^)

**Table:**

	*U*^11^	*U*^22^	*U*^33^	*U*^12^	*U*^13^	*U*^23^
Br1	0.0364 (2)	0.0205 (2)	0.0333 (2)	0.00951 (17)	0.02579 (19)	0.01161 (17)
Br2	0.0408 (3)	0.0259 (2)	0.0337 (2)	0.01040 (18)	0.0248 (2)	0.01486 (19)
Br3	0.0391 (3)	0.0254 (2)	0.0270 (2)	0.00873 (18)	0.01907 (19)	0.00274 (17)
S1	0.0331 (5)	0.0168 (4)	0.0233 (4)	0.0063 (4)	0.0191 (4)	0.0041 (4)
O2	0.0382 (16)	0.0165 (13)	0.0269 (14)	0.0053 (11)	0.0206 (12)	0.0053 (11)
O4	0.0318 (16)	0.0381 (18)	0.0357 (17)	0.0024 (13)	0.0237 (14)	0.0115 (14)
O1	0.0374 (16)	0.0212 (14)	0.0280 (14)	0.0086 (12)	0.0232 (13)	0.0034 (12)
O3	0.0298 (14)	0.0230 (14)	0.0316 (15)	0.0018 (11)	0.0181 (12)	0.0050 (12)
O6	0.0329 (15)	0.0240 (14)	0.0307 (15)	0.0037 (12)	0.0210 (13)	0.0015 (12)
O5	0.0376 (17)	0.0455 (19)	0.0226 (15)	0.0078 (14)	0.0183 (13)	0.0051 (14)
N1	0.0297 (17)	0.0204 (16)	0.0213 (16)	0.0050 (13)	0.0182 (13)	0.0047 (13)
N2	0.0299 (17)	0.0273 (18)	0.0249 (17)	0.0086 (14)	0.0183 (14)	0.0049 (14)
C20	0.028 (2)	0.027 (2)	0.034 (2)	0.0084 (16)	0.0218 (17)	0.0130 (17)
C19	0.030 (2)	0.0172 (18)	0.0255 (19)	0.0051 (15)	0.0158 (16)	0.0057 (15)
C2	0.031 (2)	0.0206 (18)	0.027 (2)	0.0091 (15)	0.0207 (17)	0.0091 (16)
C9	0.035 (2)	0.0190 (18)	0.0243 (19)	0.0096 (16)	0.0176 (16)	0.0032 (15)
C8	0.0286 (19)	0.0233 (19)	0.027 (2)	0.0075 (16)	0.0183 (16)	0.0058 (16)
C10	0.039 (2)	0.0204 (19)	0.030 (2)	0.0084 (17)	0.0221 (18)	0.0064 (17)
C24	0.035 (2)	0.023 (2)	0.026 (2)	0.0053 (16)	0.0196 (17)	0.0060 (16)
C16	0.033 (2)	0.0178 (17)	0.0229 (18)	0.0071 (15)	0.0210 (16)	0.0050 (15)
C15	0.033 (2)	0.0175 (18)	0.0235 (18)	0.0068 (15)	0.0199 (16)	0.0058 (15)
C21	0.036 (2)	0.031 (2)	0.026 (2)	0.0092 (18)	0.0232 (18)	0.0101 (17)
C7	0.029 (2)	0.024 (2)	0.038 (2)	0.0053 (16)	0.0234 (18)	0.0085 (18)
C4	0.030 (2)	0.0198 (18)	0.0259 (19)	0.0078 (15)	0.0186 (16)	0.0075 (15)
C3	0.0298 (19)	0.0206 (18)	0.027 (2)	0.0096 (15)	0.0193 (16)	0.0093 (16)
C17	0.033 (2)	0.0176 (17)	0.0259 (19)	0.0069 (15)	0.0220 (17)	0.0077 (15)
C22	0.030 (2)	0.0206 (18)	0.0259 (19)	0.0059 (15)	0.0193 (16)	0.0066 (16)
C18	0.031 (2)	0.0198 (18)	0.0241 (19)	0.0069 (15)	0.0184 (16)	0.0038 (15)
C23	0.030 (2)	0.023 (2)	0.035 (2)	0.0029 (16)	0.0195 (18)	0.0078 (17)
C6	0.038 (2)	0.026 (2)	0.031 (2)	0.0103 (17)	0.0270 (18)	0.0110 (17)
C1	0.033 (2)	0.0214 (18)	0.0226 (19)	0.0074 (16)	0.0202 (16)	0.0074 (15)
C5	0.037 (2)	0.025 (2)	0.0251 (19)	0.0087 (17)	0.0216 (17)	0.0070 (16)
C11	0.052 (3)	0.025 (2)	0.036 (2)	0.015 (2)	0.026 (2)	0.0113 (19)
C14	0.041 (2)	0.0208 (19)	0.035 (2)	0.0069 (17)	0.026 (2)	0.0049 (17)
C12	0.047 (3)	0.032 (2)	0.047 (3)	0.019 (2)	0.024 (2)	0.011 (2)
C13	0.038 (2)	0.036 (3)	0.057 (3)	0.013 (2)	0.030 (2)	0.013 (2)
Cl2	0.154 (4)	0.0441 (15)	0.0602 (18)	0.0185 (18)	0.049 (2)	0.0213 (13)
Cl3	0.079 (2)	0.103 (3)	0.124 (4)	0.015 (2)	0.039 (2)	0.051 (3)
Cl1	0.175 (4)	0.082 (2)	0.0621 (17)	0.085 (2)	0.091 (2)	0.0492 (16)
C1A	0.152 (14)	0.051 (6)	0.044 (6)	0.021 (8)	0.054 (8)	0.014 (5)

### Geometric parameters (Å, º)

**Table:**

Br1—C16	1.978 (4)	C16—H16	1.0000
Br2—C15	1.974 (4)	C16—C15	1.542 (5)
Br3—C24	1.971 (4)	C16—C17	1.519 (5)
S1—O2	1.429 (3)	C15—H15	1.0000
S1—O1	1.430 (3)	C15—C1	1.498 (5)
S1—N1	1.676 (3)	C21—H21	0.9500
S1—C9	1.754 (4)	C21—C22	1.415 (5)
O4—C20	1.369 (5)	C7—H7	0.9500
O4—C23	1.429 (6)	C7—C6	1.399 (6)
O3—C19	1.362 (5)	C4—C3	1.392 (6)
O3—C23	1.443 (5)	C4—C5	1.401 (5)
O6—N2	1.236 (5)	C17—C22	1.399 (5)
O5—N2	1.227 (5)	C17—C18	1.406 (6)
N1—C4	1.427 (5)	C18—H18	0.9500
N1—C1	1.423 (5)	C23—H23A	0.9900
N2—C22	1.459 (5)	C23—H23B	0.9900
C20—C19	1.387 (6)	C6—H6	0.9500
C20—C21	1.365 (6)	C6—C5	1.394 (6)
C19—C18	1.376 (5)	C5—H5	0.9500
C2—C24	1.494 (6)	C11—H11	0.9500
C2—C3	1.436 (5)	C11—C12	1.392 (7)
C2—C1	1.371 (6)	C14—H14	0.9500
C9—C10	1.389 (6)	C14—C13	1.378 (7)
C9—C14	1.390 (6)	C12—H12	0.9500
C8—H8	0.9500	C12—C13	1.392 (7)
C8—C7	1.388 (6)	C13—H13	0.9500
C8—C3	1.400 (6)	Cl2—C1A	1.609 (12)
C10—H10	0.9500	Cl3—C1A	1.815 (18)
C10—C11	1.380 (7)	Cl1—C1A	1.742 (15)
C24—H24A	0.9900	C1A—H1A	1.0000
C24—H24B	0.9900		
			
O2—S1—O1	120.07 (17)	C22—C21—H21	121.9
O2—S1—N1	106.78 (17)	C8—C7—H7	119.7
O2—S1—C9	109.47 (19)	C8—C7—C6	120.7 (4)
O1—S1—N1	105.88 (17)	C6—C7—H7	119.7
O1—S1—C9	109.04 (19)	C3—C4—N1	107.0 (3)
N1—S1—C9	104.44 (18)	C3—C4—C5	122.4 (4)
C20—O4—C23	105.7 (3)	C5—C4—N1	130.5 (4)
C19—O3—C23	105.4 (3)	C8—C3—C2	131.0 (4)
C4—N1—S1	125.3 (3)	C4—C3—C2	108.6 (4)
C1—N1—S1	126.2 (3)	C4—C3—C8	120.4 (4)
C1—N1—C4	107.8 (3)	C22—C17—C16	124.1 (4)
O6—N2—C22	118.8 (3)	C22—C17—C18	118.3 (4)
O5—N2—O6	122.9 (4)	C18—C17—C16	117.4 (3)
O5—N2—C22	118.2 (3)	C21—C22—N2	114.4 (3)
O4—C20—C19	109.9 (4)	C17—C22—N2	122.5 (3)
C21—C20—O4	128.3 (4)	C17—C22—C21	123.1 (4)
C21—C20—C19	121.7 (4)	C19—C18—C17	118.2 (4)
O3—C19—C20	110.1 (3)	C19—C18—H18	120.9
O3—C19—C18	127.6 (4)	C17—C18—H18	120.9
C18—C19—C20	122.3 (4)	O4—C23—O3	107.8 (3)
C3—C2—C24	123.9 (4)	O4—C23—H23A	110.1
C1—C2—C24	128.1 (3)	O4—C23—H23B	110.1
C1—C2—C3	108.0 (4)	O3—C23—H23A	110.1
C10—C9—S1	118.3 (3)	O3—C23—H23B	110.1
C10—C9—C14	121.9 (4)	H23A—C23—H23B	108.5
C14—C9—S1	119.8 (3)	C7—C6—H6	118.8
C7—C8—H8	121.0	C5—C6—C7	122.3 (4)
C7—C8—C3	118.1 (4)	C5—C6—H6	118.8
C3—C8—H8	121.0	N1—C1—C15	122.1 (4)
C9—C10—H10	120.6	C2—C1—N1	108.5 (3)
C11—C10—C9	118.8 (4)	C2—C1—C15	129.2 (4)
C11—C10—H10	120.6	C4—C5—H5	122.0
Br3—C24—H24A	109.3	C6—C5—C4	116.0 (4)
Br3—C24—H24B	109.3	C6—C5—H5	122.0
C2—C24—Br3	111.4 (3)	C10—C11—H11	120.0
C2—C24—H24A	109.3	C10—C11—C12	119.9 (4)
C2—C24—H24B	109.3	C12—C11—H11	120.0
H24A—C24—H24B	108.0	C9—C14—H14	120.6
Br1—C16—H16	109.5	C13—C14—C9	118.8 (4)
C15—C16—Br1	106.4 (2)	C13—C14—H14	120.6
C15—C16—H16	109.5	C11—C12—H12	119.8
C17—C16—Br1	108.4 (3)	C13—C12—C11	120.5 (5)
C17—C16—H16	109.5	C13—C12—H12	119.8
C17—C16—C15	113.4 (3)	C14—C13—C12	120.1 (5)
Br2—C15—H15	108.9	C14—C13—H13	120.0
C16—C15—Br2	106.6 (3)	C12—C13—H13	120.0
C16—C15—H15	108.9	Cl2—C1A—Cl3	106.5 (9)
C1—C15—Br2	110.4 (3)	Cl2—C1A—Cl1	120.0 (9)
C1—C15—C16	112.9 (3)	Cl2—C1A—H1A	108.5
C1—C15—H15	108.9	Cl3—C1A—H1A	108.5
C20—C21—H21	121.9	Cl1—C1A—Cl3	104.4 (7)
C20—C21—C22	116.2 (4)	Cl1—C1A—H1A	108.5
			
Br1—C16—C15—Br2	178.14 (17)	C10—C9—C14—C13	−0.2 (7)
Br1—C16—C15—C1	56.7 (4)	C10—C11—C12—C13	−0.3 (8)
Br1—C16—C17—C22	−118.0 (4)	C24—C2—C3—C8	−4.9 (7)
Br1—C16—C17—C18	66.7 (4)	C24—C2—C3—C4	177.2 (4)
Br2—C15—C1—N1	119.7 (4)	C24—C2—C1—N1	−175.3 (4)
Br2—C15—C1—C2	−65.5 (5)	C24—C2—C1—C15	9.4 (7)
S1—N1—C4—C3	−168.6 (3)	C16—C15—C1—N1	−121.0 (4)
S1—N1—C4—C5	13.3 (6)	C16—C15—C1—C2	53.7 (6)
S1—N1—C1—C2	168.0 (3)	C16—C17—C22—N2	10.0 (6)
S1—N1—C1—C15	−16.3 (6)	C16—C17—C22—C21	−171.8 (4)
S1—C9—C10—C11	−178.7 (3)	C16—C17—C18—C19	174.1 (4)
S1—C9—C14—C13	179.3 (4)	C15—C16—C17—C22	124.1 (4)
O2—S1—N1—C4	139.8 (3)	C15—C16—C17—C18	−51.3 (5)
O2—S1—N1—C1	−30.3 (4)	C21—C20—C19—O3	−176.2 (4)
O2—S1—C9—C10	179.9 (3)	C21—C20—C19—C18	3.4 (7)
O2—S1—C9—C14	0.4 (4)	C7—C8—C3—C2	−176.6 (4)
O4—C20—C19—O3	1.0 (5)	C7—C8—C3—C4	1.1 (6)
O4—C20—C19—C18	−179.4 (4)	C7—C6—C5—C4	1.1 (6)
O4—C20—C21—C22	−178.1 (4)	C4—N1—C1—C2	−3.5 (4)
O1—S1—N1—C4	10.7 (4)	C4—N1—C1—C15	172.2 (4)
O1—S1—N1—C1	−159.3 (3)	C3—C2—C24—Br3	73.2 (5)
O1—S1—C9—C10	−47.0 (4)	C3—C2—C1—N1	2.6 (5)
O1—S1—C9—C14	133.6 (3)	C3—C2—C1—C15	−172.7 (4)
O3—C19—C18—C17	177.7 (4)	C3—C8—C7—C6	0.1 (6)
O6—N2—C22—C21	−150.6 (4)	C3—C4—C5—C6	0.1 (6)
O6—N2—C22—C17	27.7 (6)	C17—C16—C15—Br2	−62.8 (4)
O5—N2—C22—C21	26.3 (5)	C17—C16—C15—C1	175.8 (3)
O5—N2—C22—C17	−155.4 (4)	C22—C17—C18—C19	−1.5 (6)
N1—S1—C9—C10	65.9 (4)	C18—C17—C22—N2	−174.7 (4)
N1—S1—C9—C14	−113.6 (3)	C18—C17—C22—C21	3.5 (6)
N1—C4—C3—C2	−1.4 (4)	C23—O4—C20—C19	5.7 (5)
N1—C4—C3—C8	−179.5 (4)	C23—O4—C20—C21	−177.4 (4)
N1—C4—C5—C6	178.0 (4)	C23—O3—C19—C20	−7.1 (4)
C20—O4—C23—O3	−10.0 (4)	C23—O3—C19—C18	173.3 (4)
C20—C19—C18—C17	−1.8 (6)	C1—N1—C4—C3	2.9 (4)
C20—C21—C22—N2	176.4 (4)	C1—N1—C4—C5	−175.1 (4)
C20—C21—C22—C17	−1.9 (6)	C1—C2—C24—Br3	−109.2 (4)
C19—O3—C23—O4	10.5 (4)	C1—C2—C3—C8	177.1 (4)
C19—C20—C21—C22	−1.5 (6)	C1—C2—C3—C4	−0.8 (5)
C9—S1—N1—C4	−104.3 (3)	C5—C4—C3—C2	176.9 (4)
C9—S1—N1—C1	85.7 (4)	C5—C4—C3—C8	−1.3 (6)
C9—C10—C11—C12	−0.5 (7)	C11—C12—C13—C14	0.9 (8)
C9—C14—C13—C12	−0.6 (7)	C14—C9—C10—C11	0.8 (6)
C8—C7—C6—C5	−1.3 (7)		

### Hydrogen-bond geometry (Å, º)

**Table:**

*D*—H···*A*	*D*—H	H···*A*	*D*···*A*	*D*—H···*A*
C8—H8···O5^i^	0.95	2.58	3.525 (6)	170
C13—H13···Cl13^ii^	0.95	2.63	3.244 (8)	123
C14—H14···O2^iii^	0.95	2.57	3.518 (6)	172
C1*A*—H1*A*···O1^iv^	1.00	2.34	3.169 (15)	140
C5—H5···O1	0.95	2.32	2.865 (6)	116
C15—H15···O2	1.00	2.30	2.950 (5)	122
C16—H16···O6	1.00	2.16	2.832 (5)	123
C24—H24*B*···Br2	0.99	2.83	3.572 (4)	132

Symmetry codes: (i) −*x*+2, −*y*+1, −*z*+2; (ii) −*x*, −*y*, −*z*; (iii) −*x*+1, −*y*, −*z*+1; (iv) −*x*+1, −*y*, −*z*.
